# Correction: Unusual reversibility in molecular break-up of PAHs: the case of pentacene dehydrogenation on Ir(111)

**DOI:** 10.1039/d0sc90287j

**Published:** 2021-01-08

**Authors:** Davide Curcio, Emil Sierda, Monica Pozzo, Luca Bignardi, Luca Sbuelz, Paolo Lacovig, Silvano Lizzit, Dario Alfè, Alessandro Baraldi

**Affiliations:** Department of Physics, University of Trieste Via Valerio 2 34127 Trieste Italy alessandro.baraldi@elettra.eu +39 040 3758719; Department of Physics, University of Hamburg Jungiusstrasse 11 D-20355 Hamburg Germany; Institute of Physics, Poznan University of Technology Piotrowo 3 60-965 Poznan Poland; Department of Earth Sciences, Thomas Young Center, University College London 5 Gower Place London WC1E 6BS UK; London Centre for Nanotechnology, Thomas Young Centre, University College London 17-19 Gordon Street London WC1H 0AH UK; Elettra-Sincrotrone Trieste S.C.p.A. Strada Statale 14 – km 163.5 in AREA Science Park 34149 Trieste Italy; Dipartimento di Fisica “Ettore Pancini”, Università di Napoli “Federico II” Monte S. Angelo 80126 Napoli Italy; IOM-CNR, Laboratorio TASC Strada Statale 14 – km 163.5 in AREA Science Park 34149 Trieste Italy

## Abstract

Correction for ‘Unusual reversibility in molecular break-up of PAHs: the case of pentacene dehydrogenation on Ir(111)’ by Davide Curcio *et al.*, *Chem. Sci.*, 2021, DOI: 10.1039/d0sc03734f.

The authors regret the omission of a funding acknowledgement in the original article. This acknowledgement is given below.

E. S. acknowledges financing from Polish budget funds for science in 2014–2017 as a research project in the program “Diamond Grant” No. 0084/DIA/2014/43.

The Royal Society of Chemistry apologises for these errors and any consequent inconvenience to authors and readers.

## Supplementary Material

